# Postural sway in the moving room scenario: New evidence for functional dissociation between self-motion perception and postural control

**DOI:** 10.1371/journal.pone.0257212

**Published:** 2021-09-10

**Authors:** Kentaro Horiuchi, Kuniyasu Imanaka, Masami Ishihara

**Affiliations:** 1 Department of Human Sciences, Tokyo Metropolitan University, Tokyo, Japan; 2 Department of Health Promotion Sciences, Tokyo Metropolitan University, Tokyo, Japan; Tokai University, JAPAN

## Abstract

Postural control in quiet standing is often explained by a reflexive response to optical flow, the apparent motion of environmental objects in a visual scene. However, moving room experiments show that even small-amplitude body sway can evoke odd sensations or motion sickness, indicating that a consciousness factor may also be involved. Studies targeting perception of self-motion, vection, typically use rapid visual stimuli moving in a single direction to maintain a constant feeling of vection, and there are few studies of vection using low-speed sinusoidal visual stimuli similar to human pendular movement. In the present study we searched for changes in postural control during periods of vection during quiet standing. Participants (N = 19, age = 20.4 ±1.1 years) were shown dynamic visual stimuli in the form of sinusoidally expanding and contracting random dots, and the stimuli speed and visual field were manipulated. Posture was continually evaluated using Center of Pressure (CoP) measurements. Participants were also asked to report feelings of vection, both by pressing a button during the trial and through an overall rating at the end of each trial. Using repeated-measures ANOVA, we assessed changes in the CoP and vection variables between experimental conditions, as well as possible interactions between the variables. The results show that postural reaction and vection were both affected by the visual stimuli and varied with speed. The peripheral visual field was found to couple to stronger feeling of vection and better quality of postural control. However, no significant relationship between postural control and vection, nor evidence of vection interaction to the relationship between optical flow and postural control, was found. Based on our results we conclude that for postural stability during quiet standing, visual cues dominate over any potential consciousness factor arising due to vection.

## Introduction

Postural control during quiet standing is complex and requires interaction between many perceptual and motor processes, with visual cues from the environment having been shown to play an important role [1]. In performing stable quiet standing, several types of sensory input—primarily visual, somatosensory and vestibular—depend on the external environment as well as internal body changes, and is integrated into a multi-sensory scenario by the central nervous system [2]. This information is then further processed and used to produce motor signals, creating coordinated reflexive muscle activity to maintain postural control. In case of conflicting sensory stimuli, sensory reweighting—where more reliability is given to one sensory contribution than others—occurs in real time to accommodate environmental changes [3]. Vision is recognized as one of the most important sources of information in postural control during quiet standing, often overriding other sensory input such as the vestibular or the somatosensory [4].

The important role of vision in postural control during quiet standing is often explained by a reflexive response to optical flow, the retinal motion of environmental objects in a visual scene [5, 6]. In quiet standing people naturally sway to compensate for small postural deviations to either side [7, 8]. Sway in the anterior-posterior direction is bigger than medial-lateral owing to the configuration of the ankle joint and orientation of the head and body [9, 10]. The classical moving room experiment by Lishman & Lee (1973) was designed to study this reflexive postural control: participants stood on a stable floor surrounded by three-way walls and a ceiling that were slowly oscillated in a forward and backward direction by the experimenter, giving anterior-posterior visual cues similar to small-amplitude postural sway [11]. The resulting movement pattern of participants showed anterior-posterior displacement matching the frequency of the moving room. This result has since been replicated in numerous experimental setups [3, 12–14], and the induced postural sway can be understood in terms of a natural postural response to reduce optical flow. Efficient postural stability during quiet standing requires the body to move as little as possible, and under ordinary static standing conditions the amount of optical flow can be seen as a measure of how much the body moves. Indeed, several studies indicate that postural control aims to minimize optical flow [5, 6, 11, 15]. In a setting where the optical flow is experimentally manipulated, such as a moving room, participants standing still will reflexively adjust their posture to minimize the flow, thereby creating body sway synchronized with the dynamic stimuli. In such artificial conditions, the natural motor reaction to minimize optical flow will thus create more movement rather than stabilize posture; nevertheless, the visual stimuli still dominate the postural response. The results of moving room experiments are well explained within the framework of reducing optical flow, and allow for the study of quiet standing slow postural sway in near real-world conditions.

In moving room experiments participants typically do not consciously perceive the movement of the room, yet there are often reports of odd sensations or motion sickness [14, 15]. These feelings indicate that there may be a conscious factor in postural control, in addition to the reflexive (subconscious) one, even during quiet standing. While both subconscious and conscious response patterns are mediated via the central nervous system, the neural pathways likely differ [16]. Subconscious postural control primarily involves the brain stem and spinal cord (online control; [17]), whereas cognitive information of the self-body and environment requires processing in the pre-frontal cortex and cerebellum [18]. However, studies have shown that the cerebral cortex is also involved in reflexive balance control during quiet standing [19], raising the possibility of interaction between conscious and subconscious responses. For example, awareness of self-motion (vection) may affect the postural response to optical flow (i.e., reflexive action). Determining whether such an interaction exists will give new insight to the conscious-subconscious relationship during postural control in static standing.

Investigating the potential role of consciousness in quiet standing requires a setup with low-amplitude, low-frequency conditions. Previous studies have shown that vection can impact postural response [20–24]; however, the adopted visual-stimulus speed was high in order to create conditions where vection would be evoked and constant speed or direction (contraction, expansion, rotation) were used to maintain a continuous feeling of vection. Such rapid visual stimuli lie far outside the range of natural human motion, and therefore evoke an acute, high-alert body reaction. There have been very few studies exploring a possible role of self-motion perception in postural control under conditions similar to the moving room framework (e.g., using sinusoidally expanding and contracting stimuli, with small amplitude so participants are not aware of their body sway) necessary to test the impact of vection on reflexive, small-amplitude body sway.

There are several visual factors that are known to affect postural control, in particular the visual field and the amount of optical flow. The peripheral visual field is known to have an advantage for postural control [10, 25] which may be coupled to a greater sensitivity or stronger response to vection. Furthermore, vection has been shown to vary with the speed of visual stimuli [26, 27]. In the context of oscillating stimuli, greater amplitude (and thereby greater speed) is therefore likely associated with larger vection. However, it is not known which speeds are required to evoke vection, and such a threshold may also vary between individuals.

In the present study we examined the potential impact of vection on the relationship between optical flow and postural control during quiet standing, using conditions simulating the body leaning forward and backward in a small-scale motion. Our hypothesis was that postural stability during quiet standing would be modified in the presence of vection, leading to measurable changes in postural sway. Any such effect is expected to be strongest for large-amplitude stimuli in the peripheral visual field. To test this we presented sinusoidally expanding and contracting on-screen stimuli, experimentally controlling for amplitude and the visual field (central and peripheral visual field). The trajectory of postural sway was measured using the center of pressure (CoP), and vection was evaluated in real time throughout each trial, allowing us to compare vection properties (onset, total duration, overall strength) and postural sway between participants. In order to make our results transferrable to real-world standing conditions, both amplitude and frequency of the stimuli are chosen to lie close to natural values. However, our setup is designed to produce vection and explore the postural response to sinusoidally oscillating visual stimuli rather than mimic natural body sway.

## Materials and methods

### Participants

A power analysis (G*Power 3.1, [28]) showed that our study design required 15 participants to reach a power of at least 0.80. 19 undergraduate students [(15 male and 4 female), aged 20.4 ±1.1year, height 1.73 ±0.09 m, weight 61.34 ±11.55 kg] joined this experiment. They all had normal or corrected-to-normal visual acuity, assessed via the Landolt ring test. All participants reported having no other visual impairment, such as deficiency in visual field or presence of diplopia. In addition, there was no evidence or known history of postural, vestibular or neurological impairments. All participants were naive to experiments of using dynamic visual stimuli or vection, and had never tried virtual reality or virtual environment experiments. The experiment was approved by the Ethics Committee of Tokyo Metropolitan University, and each participant was informed of the experimental procedures and gave written consent to take part in the experiment.

### Apparatus

#### Experimental room

The experiment was conducted in a dark room. Light from outside the room was occluded, and all the apparatus emitting light in the dark room were covered with a black sheet. The edge of the display was not visible in the dark room, and no feedback reporting that the edge was seen was received. For the participants, only the presented visual stimuli were seen and no other visual cues were available. The inside of the dark room was monitored using an infrared camera during the experimental trials by the experimenter seated outside the room.

#### Force plate and button

A Nintendo Wii Fit board and Nintendo Wii remote controller were used to measure the trajectory of CoP and button pressing during quiet standing. All data were input to a personal computer (through Bluetooth) using free software (OSCulator, http://osculator.net/). The nominal sampling rate for both Wii Fit board and remote is 100 Hz, but has been shown to be unstable, up to 40% of the time dropping to 54 Hz [29]. Off-line, all data were therefore reprocessed to 100Hz sampling through linear interpolation and a 20Hz Butterworth low-pass filter was applied. Any delay introduced by the Bluetooth transmission was determined to be lower than the timing accuracy of the measurements (1 ms). Although the Wii Fit board is originally designed for gaming, the accurate sensor and portability have led to it being used in several studies as a force plate to measure quiet standing [30, 31]. The reliability for such studies has been verified [29, 32–34], and the application was tested in our pilot study [35].

Full characterization of human posture is complex and there are many parameters to consider, such as joint movement, center of gravity and 3D motion [36]. However, for upright standing a simplified approach using the horizontal CoP displacement is the common approach in modeling of postural stability [37]. In this study, two CoP dependent variables, Y-axis (anterior-posterior) range of the CoP trajectory and total length of CoP trajectory (cm), were calculated from the CoP trajectory data to analyze postural control both quantitatively and qualitatively. The resulting model corresponds to a mathematical inverted pendulum, which is still considered as the scientific consensus today. The Y-axis range of the CoP trajectory represents the motion of the foot joint and how big the postural sway was in a quantitative way. We focus on the anterior-posterior motion, as the presented visual stimuli simulate postural motion along this axis only, and the participants are viewing the display face-on. The total length of CoP shows how much effort a participant needs to adjust to the dynamic visual stimuli in order to keep their posture stable, allowing us to understand the participants’s sway in a qualitative sense. A low value of this variable means more effective postural control.

#### Definition of variables

The equations of the variables are shown below, using the definitions:

$$\text{X}=\{{\text{X}}_{1},{\text{X}}_{2},\ldots {\text{X}}_{\text{N}}\}$$

$$\text{Y}=\{{\text{Y}}_{1},{\text{Y}}_{2},\ldots {\text{Y}}_{\text{N}}\}$$

N = number of data points

*Y-axis range of CoP trajectory (cm)*. The difference between the maximum and minimum values of the data points.

$$L_{Y}=Max_{\left(Y\right)}-Min_{\left(Y\right)}$$

L_Y_ = Y-axis range of CoP trajectory

*Total length of CoP trajectory (cm)*. The sum of trajectory between consecutive data points

$$L=\overset{N}{\underset{i=1}{\sum }}\sqrt{(X_{i+1}+X_{i}{)}^{2}+(Y_{i+1}+Y_{i}{)}^{2}}$$

L = total length of CoP trajectory

The raw data of the CoP (anterior-posterior and medial-lateral axes) were visually monitored by the experimenter after every trial, and if a large deviation of postural sway (more than 3 standard deviations) occurred, the data from that trial were discarded from subsequent analyses. Such large deviations were rare, and signify a postural shift unrelated to the visual stimuli (e.g., taking a step). In this case, a new trial was performed to give three viable trials for each condition.

For the vection evaluation, two variables, latency (sec) and duration (sec), were calculated from the information gathered via the Wii remote button. Latency is the time between the start of presenting dynamic visual stimuli until the participants to report vection (time the button was first pressed). Duration is the total time the button is pressed during each trial. Vection is by nature a subjective sensation, and the approach of button pressing during the trial and magnitude estimation methods after each session allows us to quantify it using well-established methods [20–24, 38].

#### Display

A 27-inch display (BenQ, GW2760HM) was used to present the visual stimuli. It has a resolution of 1920 × 1080 pixels and a refresh rate of 75 Hz. The viewing distance was set at 66cm for the experiment, and the screen height was adjusted for each participant so the center of the display was at eye level. The visual angle subtended by the screen was 45° and 25.5° for the horizontal and vertical axis, respectively.

### Stimuli and visual field conditions

#### Stimuli

**Fig 1:**
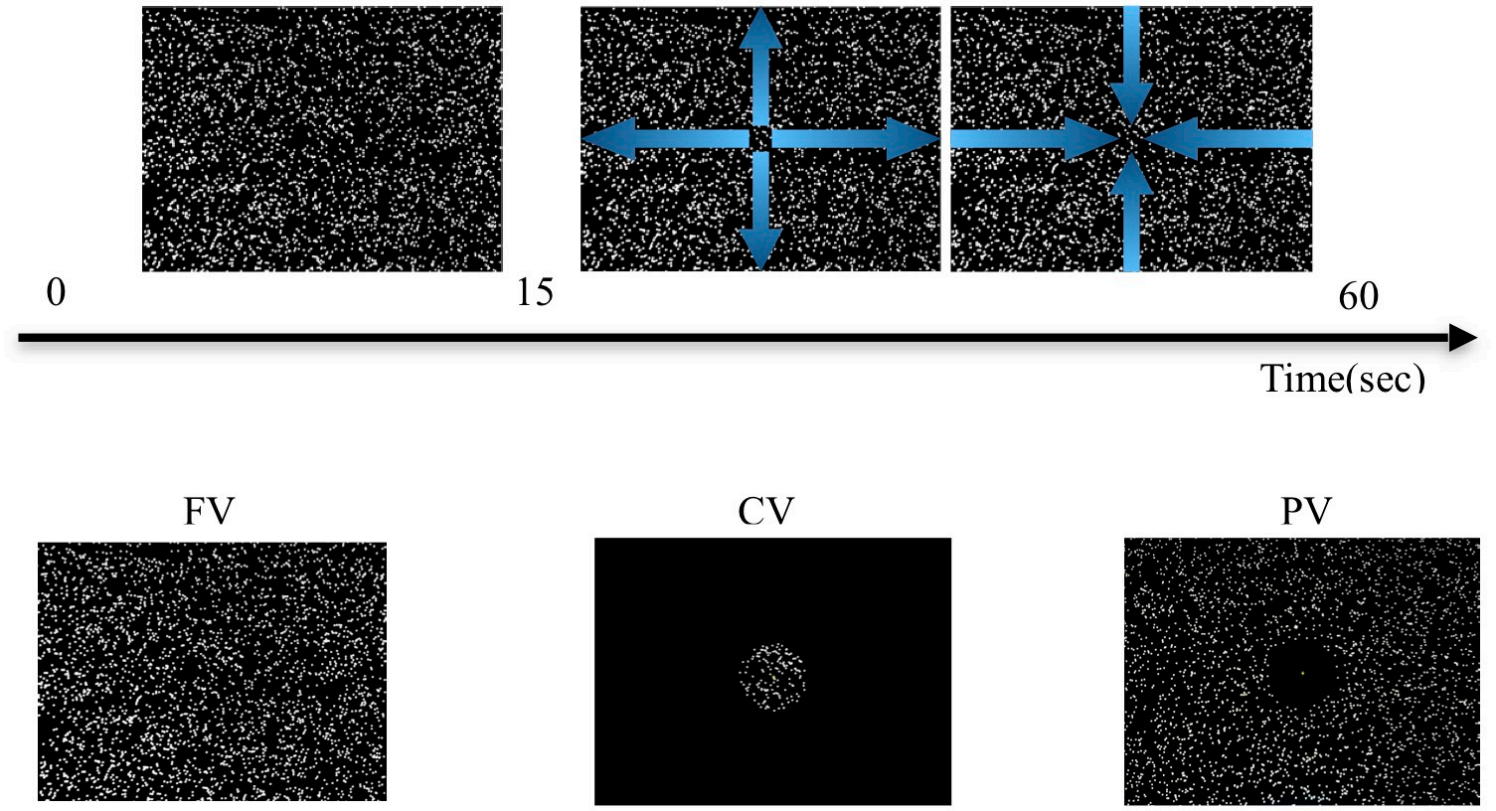
Illustration of the visual stimuli. *Top row*: Schematic showing the movement of the stimuli: first static for 15 s, then expanding and contracting for 45 s. *Bottom row*: Images show in the full vision (FV; left), central vision (CV; middle) and peripheral vision (PV; right) conditions.

The visual stimuli consisted of approximately 900 random dots (8 pixels in diameter), generated and controlled by software (Presentation, Neurobehavioral Systems) run on a computer (NUC Mini PC, Intel) and presented on the display monitor. A luminous green cross (0.68° in width and height) was used as the fixation point, presented at the center of the display monitor throughout the experimental trials. Stationary white random dots were presented on a black background for the first 15 sec, and then those dots started expanding from the center and contracting from the edge in a sinusoidal motion for 45 sec (Fig 1 and S1 Movie). The frequency of the dynamic stimuli was set to 0.08Hz. This value was chosen to be lower than the frequency of natural ankle sway (0.2–0.3 Hz; [37]), in order to more clearly separate the effect of the visual stimuli manipulation. Five levels of amplitude for the oscillating motion were set: 0, 25, 50, 100 and 200 mm. The 0 mm amplitude condition presented static random dots during the whole trial.

#### Visual field condition

The visual field conditions were experimentally manipulated into three: full vision (FV), central vision (CV) and peripheral vision (PV) conditions (Fig 1). Both the green fixation cross at the center of the display and the random dot pattern in the full display (i.e., 45° wide and 25.5° high) were presented with or without occlusion of the central or peripheral visual field. For the FV condition, the random dot pattern and fixation cross were presented with no occlusion. For the CV condition, the random dot pattern in the area outside the central visual field of approximately 8° in diameter was occluded, whereas for the PV condition the area inside the central visual field was occluded, but the fixation cross was presented.

### Procedure

The participants, who were tested individually, were asked to perform quiet standing for 60 seconds on the Wii fit board in Romberg’s standing posture (i.e., both bare feet placed side by side with no gap) and arms relaxed at either side of their body. During the trials they were asked to stare at the green fixation cross presented at their eye level in front of them throughout the trials. At the same time, the participants were also instructed to keep pressing the hand-held button of the Wii remote whenever they perceived vection in a trial. After each trial, they were asked to rate the strength of their vection experience using a magnitude estimation method. The estimated values ranged 101 points (0: no vection to 100: very strong vection). The following instruction was given to the participants regarding the button: “Please press the button while you are perceiving forward or backward self motion. If such a decision becomes difficult, or if self-motion perception disappears, please release the button.” Any more suggestion would lead to a cognitive bias about our hypothesis, because vection can be modulated by such instruction [38].

For all participants, the experiment was the first time to measure vection, so a training session (four amplitude conditions: 0, 25, 100, 200 mm) was set up to allow them to get used to the experiment. Following this, quiet standing was performed three times for each experimental condition, giving a total of 45 trials (5 levels of amplitude condition × 3 level of visual field condition × 3 trials) per participant. The orders of amplitude and visual field conditions were assigned through a randomized incomplete counterbalancing scheme, which fully considered order effects but only partially compensated for sequencing. This comprised 15 trial sequences (given 5 amplitudes and 3 visual field conditions). As 19 participants took part in the study, the last four participants were randomly assigned a sequence (all four were given different sequences). We used CoP data collected in the interval of 15 to 60 s during the 60 s standing trial for subsequent analyses. The first 15 s period of standing presented static random dots, and was considered a “settlement period”, in which a relatively large postural sway tended to occur (shown in our preliminary examination; also seen in [25]). A one-minute break was given after every trial, and a five-minute break was given after every five trials to avoid physical fatigue and prevent visual after effect. The length and timing of the rest were freely changeable by the participant for ethical reasons and to avoid motion sickness. The total experiment time was around 120 minutes. Upon completion of the whole experiment, participants were asked to write a face sheet, and then asked to verbally report their general impression about the quiet standing trials, easy/difficult amplitude or visual field conditions, etc. Subsequent categorization of the participant verbal reports showed small individual variations but no general tendencies, and there were no reports of motion sickness in any condition.

### Data analysis

The first analysis investigated if the CoP trajectory was synchronized to the sinusoidal visual stimuli (as expected in the moving room paradigm; e.g., [39]). To do this, the cross correlation between the Y-axis CoP trajectory and a 0.08Hz sine wave was computed (Wolfram Mathematica 12.0, Wolfram Research). The 0.08 Hz sine wave corresponds to the visual stimuli. The resulting cross-correlation curve was used to find the extreme value of cross correlation (either positive or negative), and the time delay at which it occurred. A positive correlation value indicates leaning forward as the pattern contracts, and a negative value instead indicates leaning backward. The time lag shows the delay in adjusting the postural sway to the optical flow (dynamic visual stimuli). A two-way (amplitude × visual field) repeated-measures ANOVA was performed for the cross-correlation value (after Fisher’s Z score processing) and the estimated time lag.

In a second step, a two-way (amplitude × visual field) repeated-measures ANOVA was performed for the CoP and vection variables, respectively. When a significant main effect or interaction was found, simple main effects tests would subsequently be performed; for significant main effects, multiple comparisons would be performed by the Bonferroni method. The statistical significance was set at α = 0.05. The Greenhouse-Geisser adjustment for significance was used when the sphericity assumption was violated. For each significant main effect, the effect size was calculated using partial eta squared ($\eta_{p}^{2}$).

To look for correlation between postural control and vection, the Pearson’s correlation coefficient between CoP variables and vection variables was computed. Both CoP and vection variables could be affected by the visual stimuli, so correlation between them were calculated at each amplitude condition. For the analysis of correlation, relative CoP variables were used; each CoP variable was normalized by the value of the 0 mm amplitude condition (static random dots condition), to account for systematic individual differences.

As a final check, participants were separated into two groups based on the total duration of vection reported. The five participants who reported the shortest vection duration were assigned to the “weak” group, and the five participants with longest vection duration to the “strong” group. Three-way mixed-design ANOVAs (vection group, amplitude and visual field) were then performed for the two CoP variables (Y-axis displacement and total CoP trajectory).

## Results

As our visual stimuli were designed to evoke anterior-posterior sway, we expected any response to be strongest in this direction. However, in our original analysis we also looked for effects on the medio-lateral displacement as well as the sway area. All variables showed the same tendencies as the anterior-posterior displacement and total length of CoP trajectory. Therefore, we only present these two variables below.

### CoP variables

**Fig 2:**
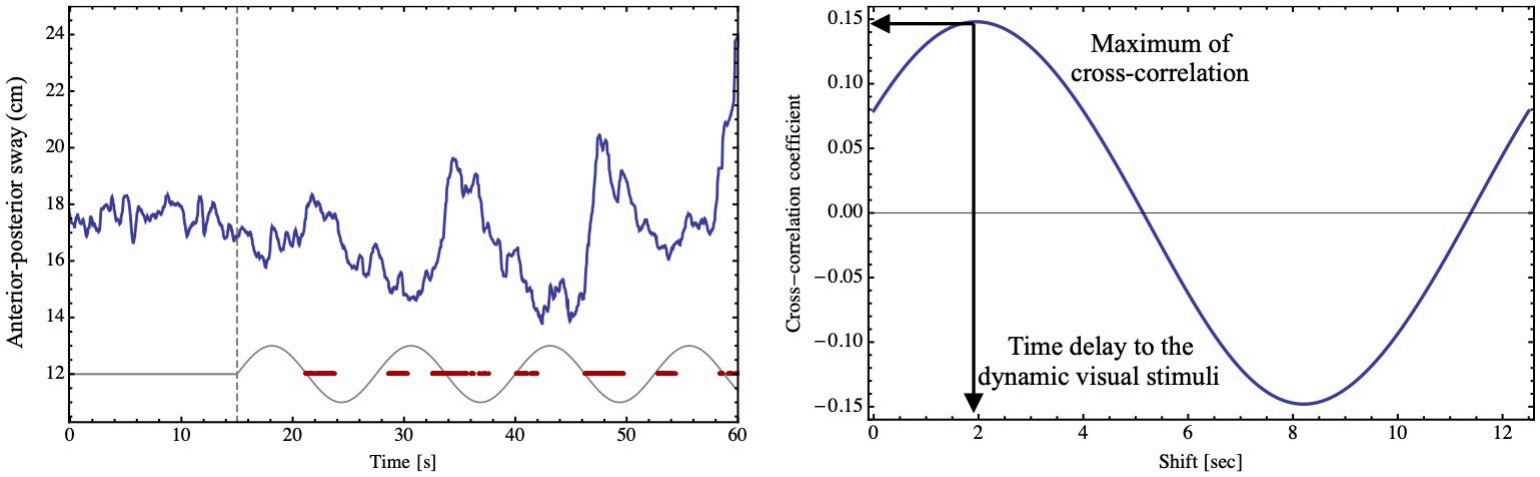
The measurement variables. *Left*: Y-axis CoP data (blue line), visual stimuli motion pattern (red line) and times that button is pressed (black). *Right*: Cross correlation between CoP and a 0.08 Hz sine wave (red line in left panel after 15 s).

A sinusoidal trend was observed in the anterior-posterior trajectory (Y- axis) of the CoP data, with a few sec delay compared to the onset of the dynamic visual stimuli (Fig 2). As described above, we computed the cross-correlation between the Y-axis motion of the CoP and a 0.08 Hz sine wave, to measure the similarity between the two time series (Fig 2). A larger cross-correlation value indicates that the anterior-posterior CoP trajectory more closely follows the sinusoidal dynamic visual stimuli. The average cross-correlation values ranged from 0.35 to 0.54 in the dynamic visual stimuli conditions, and had a mean value of 0.24 ± 0.09 for static stimuli (0 mm condition). A multiple comparison test showed that the cross-correlation value at the 0 mm amplitude condition is significantly smaller than any of the dynamic visual stimuli conditions (25, 50, 100, 200mm, *p*<0.05). A two-way (amplitude × visual field) repeated-measures ANOVA performed only including the dynamic visual stimuli showed no statistically significant differences between the amplitude conditions. A two-way (amplitude × visual field) repeated-measures ANOVA using all results shows no significant main effect for visual field [*F*(2,36) = 3.157, *p* = 0.058, $\eta_{p}^{2}=0.149$], nor any significant interaction [*F*(4.994, 89.888)<1.0]. We thus confirm that the CoP trajectories during the dynamic visual stimuli were manipulated by the visual stimuli. Analyzing the delay of lag between the CoP trajectory and the visual stimuli, there was no significant main effect for amplitude [*F*(2, 36) = 2.117, *p* = 0.087, $\eta_{p}^{2}=0.105$] or visual field [*F*(4,72) = 1.234, *p* = 0.303, $\eta_{p}^{2}=0.064$], nor any interaction [*F*(8, 144) = 1.906, *p* = 0.063, $\eta_{p}^{2}=0.096$]. The lag in the CoP trajectories was a few seconds, and similar values were seen in all visual field and dynamic amplitude conditions. Even in the big amplitude condition, the pattern of sinusoidal CoP sway and the postural reaction time to the visual stimuli were the same.

**Fig 3:**
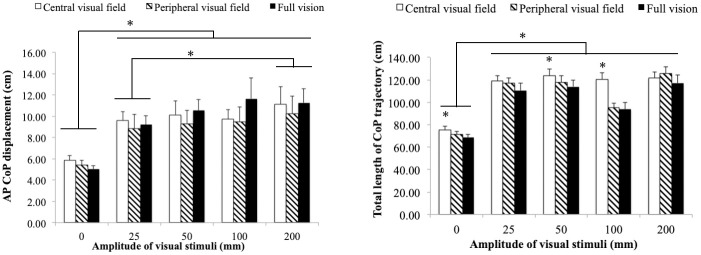
CoP variables. Anterior-posterior CoP displacement (*left*) and total length of CoP trajectories (*right*) at each amplitude. Lines with asterisks show significant differences (*p* < 0.05) between amplitude conditions, and single asterisks show significant (*p* < 0.05) differences in a visual field condition within an amplitude condition.

To test the effect of the different amplitude and visual field conditions, a two-way (amplitude × visual field) repeated-measures ANOVA was first performed for the Y-axis CoP displacement. There is a significant main effect for amplitude [*F*(1.515, 27.261) = 20.792, *p*< 0.001, $\eta_{p}^{2}=0.536$], but no significant main effect for visual field [*F*(1.278, 23.011) = 1.553, *p =* 0.230, $\eta_{p}^{2}=0.079$] or interaction [*F*(2.803, 50.449) = 2.086, *p* = 0.118, $\eta_{p}^{2}=0.104$] (Fig 3). Multiple comparisons tests show that the Y-axis CoP displacement is significantly smaller during static stimuli than the dynamic visual stimuli conditions (0mm vs 25, 50, 50, 100, 200 mm; *p* < .001). The displacement in the smallest amplitude condition was furthermore significantly smaller than in the biggest amplitude condition (25mm vs 200mm, *p* <0.05). However, the Y-axis CoP dispalcement shows no statistical difference regardless of the visual field. This indicates that both the peripheral and central visual fields are sensitive to the differences in the visual stimuli amplitude, and mediate similar postural reactions.

The total length of the CoP trajectory shows a significant main effect for both visual field [*F*(2, 36) = 12.131, *p*< 0.001, $\eta_{p}^{2}=0.403$] and amplitude [*F*(2.620, 47.152) = 138.486, *p*< 0.001, $\eta_{p}^{2}=0.885$], and also an interaction [*F*(4.330, 79.748) = 5.408, *p*< 0.001, $\eta_{p}^{2}=0.231$] (Fig 3). The subsequent simple main effects tests show a significant simple main effect for visual field in the 0 [*F*(2, 36) = 7.923, *p*< 0.001, $\eta_{p}^{2}=0.306$], 50 [*F*(2, 36) = 3.516, *p<* 0.04, $\eta_{p}^{2}=0.163$] and 100 mm [*F*(2,36) = 32.387, *p*< 0.001, $\eta_{p}^{2}=0.648$] amplitude conditions. The total length of the CoP trajectory in the static condition (0 mm) was always significantly shorter than in the dynamic conditions. At the 0, 50, and 100 mm conditions, the total length of the CoP trajectory in the CV condition was significantly longer than in the PV and FV conditions (*p* < 0.05). Postural control using the central visual field thus showed greater sway and used more energy than when using the peripheral visual field.

### Vection variables

Table 1 summarises the number of participants who perceived vection in each of the conditions (both visual field and amplitude). Although the number varied, all participants reported feeling vection in at least some conditions. Interestingly, a few participants reported vection even for the static condition.

**Table 1:** The number of participants who perceived vection at each visual field and amplitude condition.

Amplitude condition	Central visual field	Peripheral visual field	Full visual field
0mm (Static visual stimuli)	3 (15.79%)	3 (15.79%)	5 (26.32%)
25mm	13 (68.42%)	18 (94.73%)	16 (84.21%)
50mm	14 (73.68%)	18 (94.73%)	19 (100%)
100mm	18 (94.73%)	18 (94.73%)	19 (100%)
200mm	17 (89.47%)	19 (100%)	19 (100%)

**Fig 4:**
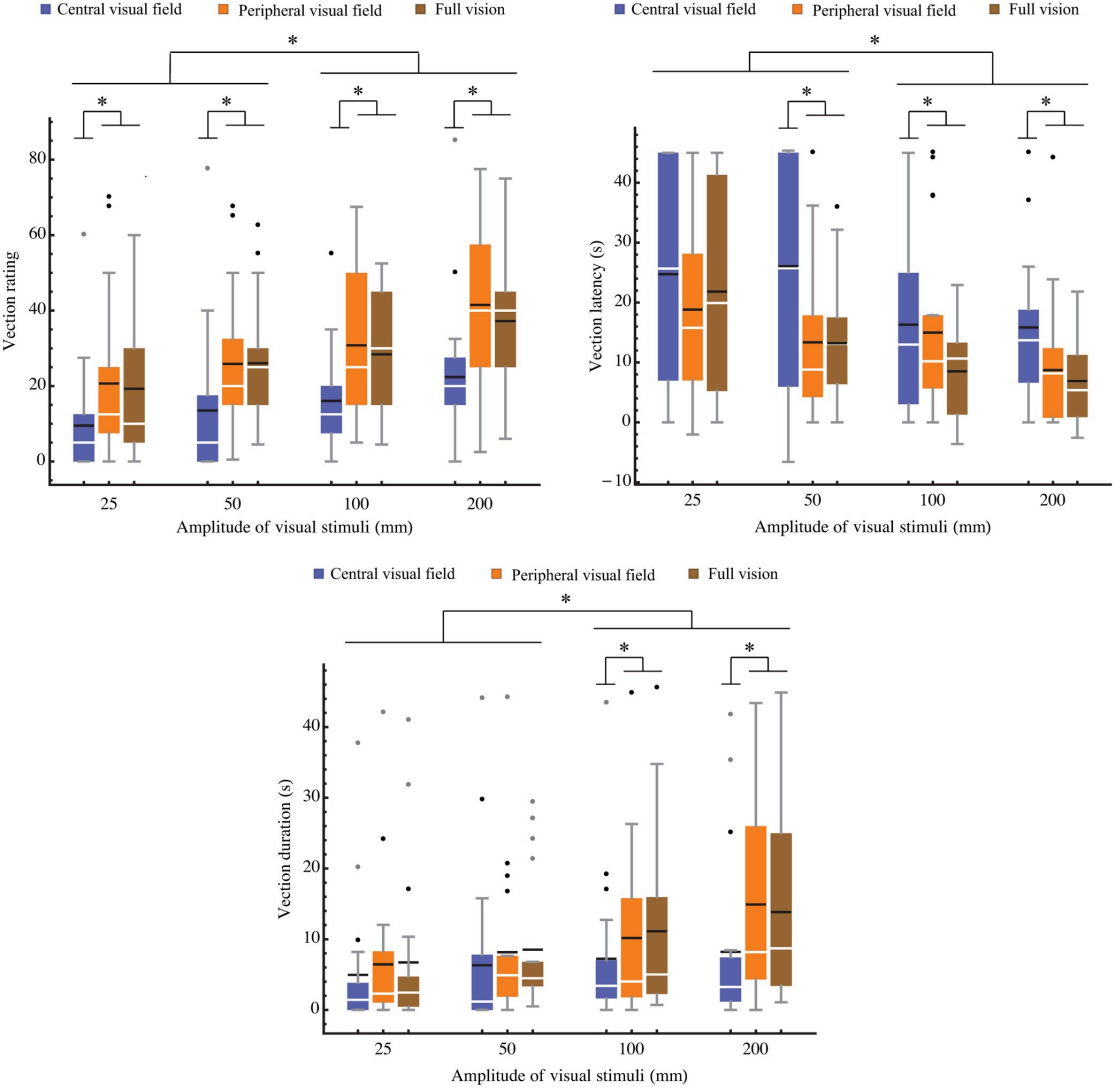
Vection variables at each amplitude. *Top left*: rating. *Top right*: latency. *Bottom*: duration. Boxplot central white line indicates the median, and the horizontal black line marks the mean. Bottom and top edges of the box indicate 25th and 75th percentiles, respectively. Whiskers show extent of data, with dots marking individual outliers. Lines with asterisks show significant differences (*p* < 0.05) between amplitude conditions, and significant (*p* < 0.05) differences between visual field conditions within an amplitude condition.

Two-way (amplitude × visual field) repeated-measures ANOVAs were then performed for the three vection variables (rating, latency, and duration), resulting in significant main effects for visual field [*F*(2, 36) = 15.050/ 12.466/ 7.847, *p<* 0.0001, $\eta_{p}^{2}=0.455/0.409/0.316$] and amplitude [*F*(3, 54) = 11.705/ 15.179/ 8.658, *p<* 0.001, $\eta_{p}^{2}=0.394/0.457/0.337$], but no significant interaction [*F*(6, 108) = 1.021/ <1.0/ 1.590, *p =* 0.416/-/ 0.158, $\eta_{p}^{2}=0.054/-/0.086$] (Fig 4). The multiple comparisons tests show that the vection rating, latency and duration in the small amplitude conditions (25, 50mm) were significantly smaller, longer and shorter, respectively, than in the large amplitude conditions (100, 200mm) (*p* < .05). Furthermore, the vection rating is significantly smaller at the central visual field condition compared to the peripheral and full visual field conditions (*p*<0.05).

### Correlation between CoP and vection variables

To search for a possible correlation between postural control and vection, Pearson’s correlation coefficient was calculated between the vection variables and the total CoP trajectory. Since both CoP and vection variables are affected by the visual stimuli, correlations were calculated at each amplitude condition. To minimize any impact caused by individual motion patterns, the values of the CoP trajectory were normalized by the value in the 0 mm amplitude condition. The results show that there is no significant correlation between postural sway and any vection variable within each amplitude condition (*p*> 0.05, Table 2).

**Table 2:** Correlation between total CoP trajectory and vection variables at each visual field condition.

Vection variables	Visual field	Amplitude of stimuli (mm)							
		25		50		100		200	
		*r* ^a^	*p* ^a^	*r*	*p*	*r*	*p*	*r*	*p*
Rating	Central visual field	0.12	0.61	0.13	0.60	0.21	0.40	0.27	0.26
	Peripheral visual field	0.04	0.87	0.24	0.33	0.11	0.65	0.22	0.37
	Full vision	0.37	0.12	0.12	0.62	0.30	0.21	0.26	0.29
Latency	Central visual field	0.00	1.00	0.16	0.51	0.37	0.12	0.07	0.79
	Peripheral visual field	0.21	0.39	0.107	0.78	0.36	0.14	0.10	0.69
	Full vision	0.17	0.49	0.27	0.26	0.04	0.86	0.29	0.23
Duration	Central visual field	0.18	0.46	0.44	0.06	0.39	0.10	0.34	0.16
	Peripheral visual field	0.06	0.82	0.05	0.83	0.06	0.81	0.01	0.98
	Full vision	0.43	0.07	0.26	0.29	0.09	0.71	0.17	0.49

^a^The correlation coefficient is denoted *r* and *p* gives the corresponding p-value.

### Comparison of “weak” and “strong” vection groups

In order to more deeply test for any coupling between vection and postural response, we separated participants into two groups based on the total duration of vection reported. We therefore assign the six participants who reported the shortest vection duration to the “weak” group, and the six participants with longest vection duration to the “strong” group. Three-way mixed-design ANOVAs (vection group, amplitude and visual field) were performed for the two CoP variables (Y-axis displacement and total CoP trajectory), and there is no significant main effect for the vection group or interaction between group and the other two factors. This result shows that although one group reported greater vection than the other, those participants did not necessarily move more.

### Possible coupling between vection and large postural sway

**Fig 5:**
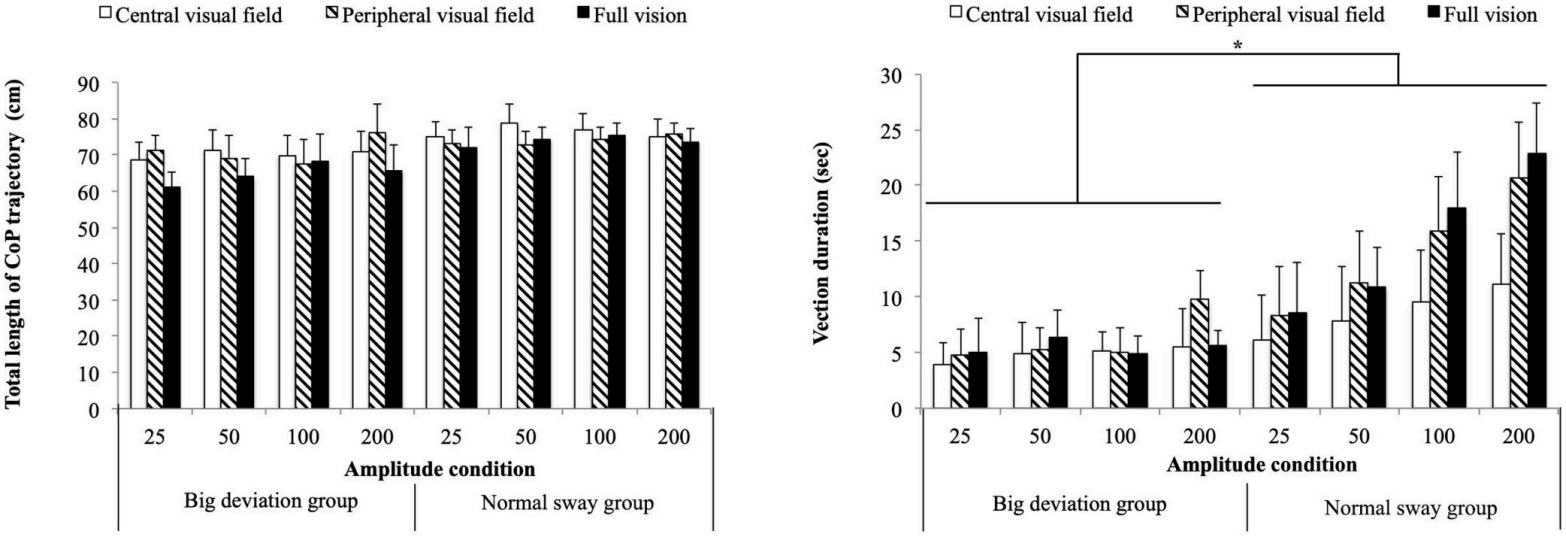
Comparison between the two sway groups. Total length of CoP trajectory (*left*) and vection duration (*bottom*) for the two groups (see text for details). Lines with asterisks show significant differences (*p* < 0.05) between amplitude conditions.

While there is a general trend of anterior-posterior motion following the dynamic visual stimuli, sometimes the participants displayed sudden episodes of greater sway, clearly above the regular random natural variations. Interestingly, in many cases vection was reported simultaneously with this deviation. The participants were therefore separated into two groups, those who showed a big (>1.5σ) CoP deviation when they reported vection (big deviation group) and those who did not (normal sway group), and three-way mixed-design ANOVAs (group, amplitude and visual field) were performed using the CoP variables. There was a significant simple main effect for the group [*F*(1, 10) = 3.445, *p =* 0.029, $\eta_{p}^{2}=0.256$], where the participants who simultaneously felt vection and swayed have significantly bigger CoP Y-axis range compared to the participants who reported vection without body sway. This is expected, as large CoP sway was a criterion for defining the groups. However, there is no significant difference in total length of CoP between the two groups [*F*(1, 10)<1.0] (Fig 5). Three-way mixed-design ANOVAs using vection variables revealed no significant main effect for the group [*F*(1, 10) = <1.0], but significant interaction for group and amplitude with vection variables [*F*(3,30) = 3.247/ 4.947/ 4.881, *p =* 0.036/ 0.018/ 0.034, $\eta_{p}^{2}=0.254/0.331/0.379$]. The subsequent simple main effect is significant only in the normal sway group [*F*(3, 15) = 9.062/ 6.262/, 3.601, *p =* 0.001/ 0.006/ 0.039, *$\eta_{p}^{2}=0.462/0.664/0.419$*], meaning that the increase of vection with amplitude was significantly larger in that group (Fig 5). For the group that showed large CoP deviations during vection, no correlation between visual stimuli amplitude and any vection variable was seen.

As this analysis was not part of the original design, no calculation of the power or required sample size was made before the experiment was conducted. A predictive (“a priori”) analysis performed with G*Power using an intermediate effect size ($\eta_{p}^{2}=0.06$), showed that 34 participants would be needed to achieve a power >0.80. However, a retrospective power analysis (“post hoc”) for the interactions based on the actual observed effect sizes ($\eta_{p}^{2}=0.245/0.331/0.379$) showed an achieved power >0.95 in all cases. Although our results are based on 12 participants, they indicate that further studies are warranted.

## Discussion

The main goal of this study was to clarify whether vection affects the role of optical flow in postural control during quiet standing. With this purpose in mind, both postural sway and vection were manipulated by the optical flow induced by the visual stimuli, and the relationship between the two factors was investigated. Sinusoidally expanding and contracting visual stimuli were used, and vection was measured both quantitatively and qualitatively. To further understand postural control and vection, possible differences between the central and peripheral visual fields were considered.

### Validation of postural manipulation and vection

Our results clearly showed that we could induce postural sway using a display with sinusoidally and slowly moving dots, as CoP variables increased with amplitude and we found the expected correlation between visual stimuli and the postural trajectory. The maximal cross-correlation values found ranged from 0.35 to 0.54, which is comparable to those found in studies using visual stimuli at frequencies close to that of natural body sway (around 0.3 Hz; e.g., [39]). We used a lower frequency to more clearly separate the effect of natural sway and the experimentally manipulated reaction, but the results nevertheless fit well with previous studies within the context of the moving room paradigm.

To further validate our setup we compared our clear-cut trends regarding vection (e.g., the relationship between vection and visual stimuli speed) to existing results. Previous vection studies have shown that vection rating, latency and duration become respectively stronger, shorter and longer when the participant feels stronger vection [26, 27]. These previous findings suggest a typical vection latency of four to seven seconds after the start of the moving stimuli [40]. We observed an average latency of 6.8 ± 1.6 sec (FV 200mm condition, where every participant reported vection), matching previous studies. We further found a proportional relationship between the amplitude of visual stimuli and vection evaluation, with vection being stronger in the bigger amplitude conditions. Previous studies have shown increasing vection evaluation with the speed of visual stimuli [26, 27], and our results agree with these findings. In studies of linear vection, dynamic visual stimuli are manipulated in one direction and/or with constant speed, while our results are obtained from stimuli where both direction and speed vary. That we nevertheless find the same major trends supports our belief that our results are indeed coupled to the same sense of vection as evoked by rapid motion, and not affected by our choice of stimuli.

Not all previous attempts to replicate a moving room have succeeded with a restricted visual field [41], but we could replicate the swinging room by using a 27-inch desktop display (45 degree visual field) in a dark room where the display was the only visual cue. Possible reasons for this include the posture of the participants (we required them to stand upright with feet together), and that the dark room made it easier to concentrate on the visual cue. Another interesting aspect in our setup which may play a role is that we asked participants to press a button during the standing trial, whenever they experienced vection. Within the constrained action hypothesis, it is possible that this multiple task created a more immersive situation, reducing the conscious focus on posture and promoting an automatic postural reaction [42, 43]. This dual task of button pressing and quiet standing could help in replicating the results of the moving room setup, even with a relatively modest visual angle.

### The relationship between postural control and vection

At first, we investigated a possible relationship between CoP and vection, and based on the correlation coefficient there is no strong evidence for an interaction between them. Within the participants there is a significant correlation between CoP and vection variables, but both variables are affected by the same dynamic visual stimuli. To investigate whether postural sway is directly affected by vection, we studied the correlation between subjects. As our results indicated, no significant correlation was found. Previous vection studies have reported that vection affects postural sway [20, 21, 44], but in these studies the speed of the visual stimuli is more than 10 times greater than in our setup. One possibility is thus that the sensation of vection might be weaker in our experiment. Nevertheless, we conclude that vection is not a mediator of postural sway under slow speed visual stimuli. Comparing with the study of Horiuchi et al. [25], our results indicate that the presence of optical flow indeed leads to more stable posture, and that vection (if present) does not have a major influence on postural stability during quiet standing.

A possible relationship between vection and postural sway was further investigated by separating the participants into “strong” and “weak” vection groups, but the postural response did not differ significantly between the groups. This result reinforces the conclusion that the amount of perceived vection does not influence postural control in slow-speed conditions, and points to large individual differences. Even if the same dynamic visual stimuli were shown, subjective self-motion perception could be different, but the feeling of vection reported cannot be coupled to postural control in our study. On a general level, both vection and postural sway increased with the visual stimuli amplitude, yet for the same visual stimuli and similar postural sway, the feeling of vection reported can vary greatly between participants.

Understanding the relationship between conscious and subconscious processing in postural control can also give information about the neural interaction behind the two processes. Visual sensory input is primarily transmitted from the retina, via the lateral geniculate nucleus (LGN) in the thalamus, to the primary visual cortex (V1). From V1 there are two major pathways: information about motion and location of objects is transmitted via the dorsal stream to the ventral intra-parietal area (VIP), and object identification and recognition is mediated via the ventral stream to the temporal lobe. There is an ongoing debate whether the functional dichotomy between dorsal and ventral streams also signify a dissociation between subconscious (dorsal) and conscious (ventral) visual processing. Increasing evidence suggests that these streams are not independent, and that there exits a cross-talk network integrating the pathways [45, 46]. For example, the VIP is known to have large receptive fields and selectivity for optical flow patterns that simulate self-motion [47], as expected for processing the spatial signals mediated by the dorsal stream. However, it has also been shown that activity in the VIP is significantly increased when experiencing vection [48], indicating its involvement during a conscious response.

If both conscious and sub-conscious responses are processed in the VIP, our results raise the question of whether there are differences in how they are mediated. The visual stimuli clearly evoke a postural reaction (subconscious) response, and for many of the trials participants report vection (signaling a conscious response). However, the presence of a conscious response does not translate to any measurable impact on postural sway. Either vection does not generate any motor signals, or they are overridden by the subconscious response. As previous studies of vection have been shown to give a clear postural response, it may be that a certain threshold is required in order for the conscious reaction to evoke a motor response. It is known that the conscious system can override the subconscious [22, 23]; our results show this need not always be the case.

Visual processing has also been connected to the extrageniculate pathway, which is a mediator of visual cues from the retina to the extrastriate cortex (the higher visual cortex). Signaling occurs through the superior colliculus (SC) and pulvinar nucleus of the thalamus, bypassing V1, and stimulus velocities have been found to be higher than that to V1 [49]. This pathway may therefore play a strong role in subconscious processing of visual signals. Although the higher visual cortex mainly depends on information from V1, the extrageniculate pathway is believed to be able to carry information about form as well as spatial perception [50]. The SC plays a major role in sensory-motor integration, further supporting a role of the pathway in postural control. In our case, the extrageniculate pathway could mediate the postural response to the visual stimuli (optical flow), whereas the sensation of vection instead is mediated via the VIP. We can then speculate that the delay in vection after presenting the visual stimuli is a sign of the different pathways. This picture also offers an explanation for the lack of change in postural stability in response to vection; although vection is generated in the VIP, these signals may require a certain threshold to be fed forward to the higher visual cortex.

Reflexive postural control is strongly coupled to the vestibular system, where signals are sent directly to the vestibular nuclei in the brain stem [51]. This can trigger a reflexive motor response through the spinal cord allowing rapid postural readjustment, before higher order processing and sensory integration. The vestibular nuclei are known to receive multisensory information, including from the visual system [52], making it relevant to consider a possible subcortical input from the visual pathways. If the same reflexive system is triggered by visual cues, this would provide further context for our finding that vection does not impact postural response—the subcortical pathways of reflexive postural adjustment allow correction before the sensory integration leading to conscious registration of vection. However, this scenario requires an additional mechanism (such as a threshold) to explain the lack of motor signals generated by vection, as postural stability remains constant throughout the trial.

In some trials vection was reported simultaneously with a large CoP deviation. These participants might have felt the real postural movement as self-motion perception, and reporting vection was therefore not a sign of visually induced vection. During the trial participants were focusing on the standing task in a dark room, so they could not discern between real movement and the self-motion illusion caused by the dynamic visual stimuli. Another possibility is that bringing self-motion perception to a conscious level evoked postural deviation. The awareness of self-motion can be seen as disturbance of the consciousness and that could affect smooth postural sway. However, visually induced vection has a few seconds latency, making it less likely that the phenomenon of simultaneous vection is related to the “typical” vection caused by the dynamic visual stimuli. The participants who simultaneously felt vection and swayed have significantly bigger CoP Y-axis range than the participants who answered vection without body sway. This is not surprising, since a period of large postural sway was a criterion used for selecting the groups. However, there was no difference in the total length of CoP, meaning that the postural strategy in both groups was the same. The analysis of vection showed an interaction between group and visual stimuli amplitude. Only the group which did not have a concurrent large sway showed the expected increase in vection with visual stimuli amplitude. We believe that this result is due to the way participants perceive the concept of vection. Some participants reported vection when feeling actual postural sway, and were therefore not so much influenced by the visual stimuli—they will always report some vection. Other participants did report visually induced vection, which then strongly correlated with the visual stimuli. Although the power analysis showed that a larger number of participants may be required to verify these findings, this is a key discovery for designing future experimental setups; one reason the two cases are mixed could be that our visual stimuli emulating slow postural motion lie close to the threshold for visually induced vection.

### Vection response and visual field dependence

In the vection variables, there were significant main effects for the visual field, and the advantage for the peripheral condition could come both from the difference of the physical area and depth information. At the peripheral visual field, the vection rating, latency, and duration were significantly stronger, shorter and longer, respectively, than that observed in the central visual field. There could be two reasons for the difference between the visual fields. One is the total area and physical quantity of visual stimuli. At the peripheral visual field, both areas and the number of dots are bigger than for the central visual field (CV: 0–7 degrees, 25 dots; PV: 7–45 degrees, 900 dots). The amount of visual stimuli presented affects the vection evaluation more than the difference between the central and the peripheral visual fields [53]. So the bigger visual field area could be the reason why vection evaluation is greater in the peripheral visual field condition. Another reason is the rich information of depth in the peripheral visual field, and the depth information makes vection evaluation stronger [54]. In our visual stimuli, the fixation cross was located in front and center, and the peripheral visual field is relatively far and back, causing binocular disparity. Presenting stimuli in the peripheral visual field could therefore lead to stronger vection because of the sense of depth. We are planning a subsequent study where the same physical quantity of visual stimuli is presented in the central and peripheral visual fields (25 dots shown in each visual field condition). Further research will reveal whether the differences in vection between visual fields is caused by our experimental setting or other factors.

The result of the total length of CoP trajectory points to a more efficient peripheral visual field also regarding postural stability. As is expected, there was a main effect for the visual field factor, and the subsequent post hoc test revealed significantly greater length in the central visual field condition than for the peripheral and full visual fields. The purpose of measuring the total length of CoP is to confirm the strategy of keeping the balance within an area. Even if participants move within the same postural area, the trajectory can be very different—many smaller movements within that area lead to a large total trajectory, while a very stable posture with a few larger deviations give a small total trajectory. Our results thus point to different strategies for or quality of visually guided postural control. Furthermore, these strategies seem not to be affected by the presence of vection. Given that the peripheral field seems to evoke stronger vection, we would expect any impact from vection on postural control to be strongest in this condition. However, we observed no effect from vection in any visual field condition, strengthening our main result that vection does not affect strategies for postural control under slow speed visual stimuli.

### Limitations

There are several ways in which our study can be improved and expanded. While CoP measurement is a common means of measuring postural control, there are a number of other measures in the literature, e.g., joint movement, center of gravity and 3D motion (e.g., [36]). Our data show no effect on the CoP, but we cannot rule out that vection can affect other measures. The discovery of is another interesting result of our study. Due to our small sample size, we cannot present any conclusive results on the two potentially different vection responses (induced by either visual stimuli or self motion), showing that a greater number of participants is needed to explore this further. A further limitation in our setup is the visual field we can investigate. Due to the size of the monitor we can only probe the near-peripheral field. The functional importance of the far peripheral field is poorly investigated [55], so presenting visual stimuli here could potentially give different results. Finally, in order to measure vection in real-time we asked participants to press a button. This effectively introduces a dual task, which could potentially affect the neural pathways and thereby influence the result of the trial. Nevertheless, we see our study as an important step towards understanding the relationship between postural control and vection.

## Conclusions

In this study we measured postural sway while presenting dynamic visual stimuli in different areas of the visual field, and investigated the dependence on the presence of vection. We presented sinusoidally expanding and contracting visual stimuli that simulate the body leaning forward and backward in small-scale naturalistic motion. We controlled on-screen stimuli with amplitude and visual field conditions. Our results showed that the visual stimuli induce a similar pattern of postural sway, with amplitude increasing with that of the visual stimuli. Vection was evaluated in real time throughout each trial, allowing us to compare vection properties (onset, total duration, overall strength) and postural sway between participants. The correlations between CoP and vection variables are not significant, and we could not support a direct relationship where participants who reported stronger vection would sway more than those reporting weak or no vection. As the presence of vection does not affect the postural sway induced by the visual stimuli, we find no evidence for interaction between conscious and subconscious response. To our knowledge there is no previous study where vection was quantitatively measured using sinusoidally expanding and contracting visual stimuli, and this study is thereby the first where vection is measured with stimuli reproducing those of the moving room paradigm. We could report on the postural sway and vection induced in the moving room paradigm, and found no significant relationship between perception and action in this experiment condition.

## Supporting information

S1 Movie Example of dynamic visual stimuli shown. Full vision, 50 mm amplitude condition.
(MOV)

## References

[1] Dijkstra TM, Schöner G, Gielen CC. Temporal stability of the action-perception cycle for postural control in a moving visual environment. *Exp Brain Res*. 1994;97,477–86. doi: 10.1007/BF00241542 8187859

[2] Steindl R, Kunz K, Schrott-Fischer A, Scholtz AW. Effect of age and sex on maturation of sensory systems and balance control. *Dev Med Child Neurol* 2006;48:477–82. doi: 10.1017/S0012162206001022 16700940

[3] Moraes R, Barbosa de Freitas P, Razuk M, Barela JA. Quality of Visual Cue Affects Visual Reweighting in Quiet Standing, *PLOS ONE*. 2016, doi: 10.1371/journal.pone.0150158 26939058 PMC4777428

[4] Bronstein AM. Multisensory integration in balance control. *Handb Clin Neurol*. 2016;137,57–66. doi: 10.1016/B978-0-444-63437-5.00004-2 27638062

[5] Fujimoto K, Ashida H. Different Head-Sway Responses to Optic Flow in Sitting and Standing With a Head-Mounted Display. *Front Psychol*. 2020;11:577305. doi: 10.3389/fpsyg.2020.577305 eCollection 2020. 33123058 PMC7573131

[6] Kelly JW, Riecke B, Loomis JM, Beaks AC. Visual control of posture in real and virtual environments. *Percept Psychophys*. 2008;70(1):158–65. doi: 10.3758/pp.70.1.158 18306969

[7] Lafond D, Duarte M, Prince F. Comparison of three methods to estimate the center of mass during balance assessment. *J Biomech*. 2004;37,1421–6. doi: 10.1016/S0021-9290(03)00251-3 15275850

[8] Colbert B, Crétual A, Allard P, Delamarche P. Force-plate based computation of ankle and hip strategies from double-inverted pendulum model. *Clin Biomech*. 2006;21,427–34. 10.1016/j.clinbiomech.2005.12.003 16442676

[9] Leardini A, O’connor JJ, Catani F, Giannini S, The role of the passive structures in the mobility and stability of the human ankle joint: a literature review, *Foot Ankle Int*. 2000;21(7):602–15. doi: 10.1177/107110070002100715 10919630

[10] Berencsi A, Ishihara M, Imanaka K. The functional role of central and peripheral vision in the control of posture. *Hum Mov Sci*. 2005;24,689–709. doi: 10.1016/j.humov.2005.10.014 16337294

[11] Lishman JR, Lee DN. The autonomy of visual kinaesthesis. *Perception*. 1973;2,287–294. doi: 10.1068/p020287 4546578

[12] Polastri PF, Berela JA. Adaptive visual re-weighting in children’s postural control. *Plus One*. 2013,8(12):e82215. doi: 10.1371/journal.pone.0082215 eCollection 2013. 24324766 PMC3853149

[13] Villard SJ, Flanagan MB, Albanese GM, Stoffregen TA. Postural instability and motion sickness in a virtual moving room. *Hum Factors*. 2008;50(2),332–45. doi: 10.1518/001872008X250728 18516843 PMC4030407

[14] Hibachi P, Slobounov S, Newell K. Egomotion and vection in young and elderly adults. *Gerontology*. 2009;55(6),637–43. doi: 10.1159/000235816 19707011

[15] Lee DN, Lishman JR. Visual proprioceptive control of stance. *Journal of Human Movement Studies*. 1975;1,87–95.

[16] Takakusaki K. Functional Neuroanatomy for Posture and Gait Control. *J Mov Disord*. 2017 Jan; 10(1): 1–17. Published online 2017 Jan 18. doi: 10.14802/jmd.16062 28122432 PMC5288669

[17] Deliagina TG, Orlovsky GN, Zelenin PV, Beloozerova IN. Neural bases of postural control *Physiology*. 2006 Jun 21:216–25. doi: 10.1152/physiol.00001.2006 16714480

[18] Fujita H, Kasubuchi K, Wakata S, Hiyamizu M, Morioka S. Role of the Frontal Cortex in Standing Postural Sway Tasks While Dual-Tasking: A Functional Near-Infrared Spectroscopy Study Examining Working Memory Capacity. *Biomed Res Int*. 2016;2016:7053867. doi: 10.1155/2016/7053867 Epub 2016 Feb 3. 27034947 PMC4791508

[19] Varghese JP, Beyer KB, Williams L, Miyasike-daSilva V, McIlroy WE. Standing still: Is there a role for the cortex? *Neuroscience Letters*. 2015: 590: 18–23. doi: 10.1016/j.neulet.2015.01.055 25623039

[20] Thurrell AEI, Bronstein AM. Vection increases the magnitude and accuracy of visually evoked postural responses. *Exp Brain Res*. 2002;147,558–560. doi: 10.1007/s00221-002-1296-1 12444489

[21] Kuno S, Kawakita T, Kawakami O, Miyake Y, Watanabe S. Postural Adjustment Response to Depth Direction Moving Patterns Produced by Virtual Reality Graphics. *Jpn J Physiol*. 1999;49,417–424. doi: 10.2170/jjphysiol.49.417 10603425

[22] Tanahashi S, Ujike H, Kozawa R, Ukai K. Effects of visually simulated roll motion on vection and postural stabilization. *J Neuroeng Rehabil*. 2007;4,39. doi: 10.1186/1743-0003-4-39 17922922 PMC2169230

[23] Lubeck AJ, Bos JE, Stins JF. Framing visual roll-motion affects postural sway and the subjective visual vertical. *Atten Percept Psychophys*. 2016;78,2612–2620. doi: 10.3758/s13414-016-1150-3 27363414 PMC5110582

[24] Palmisano S, Apthorp D, Seno T, Stapley PJ. Spontaneous postural sway predicts the strength of smooth vection. *Exp Brain Res*. 2014;232, 1185–91. doi: 10.1007/s00221-014-3835-y 24449012

[25] Horiuchi K, Ishihara M, Imanaka K. The essential role of optical flow in the peripheral visual field for stable quiet standing: Evidence from the use of a head-mounted display. *PLOS ONE*. 2017. doi: 10.1371/journal.pone.0184552 28991916 PMC5633140

[26] Tamada Y, Seno T. Roles of Size, Position, and Speed of Stimulus in Vection with Stimuli Projected on a Ground Surface. *Aerosp Med Hum Perform*. 2015;86(9):794–802. doi: 10.3357/AMHP.4206.2015 26388086

[27] Keshavarz B, Philipp-Muller AE, Hemmerich W, Riecke BE, Campos JL. The effect of visual motion stimulus characteristics on vection and visually induced motion sickness. *Displays*. 2019:58:71–81. doi: 10.1016/j.displa.2018.07.005

[28] Faul F, Erdfelder E, Lang AG, Buchner A. G*Power 3: A flexible statistical power analysis program for the social, behavioral, and biomedical sciences. *Behavior Research* 2007:175–191. 10.3758/bf03193146 17695343

[29] Audiffren J, Contal E. Preprocessing the Nintendo Wii Board Signal to Derive More Accurate Descriptors of Statokinesigrams. *Sensors*. 2016;16,1208. doi: 10.3390/s16081208 27490545 PMC5017374

[30] Young W, Ferguson S, Brault S, Craig C. Assessing and training standing balance in older adults: a novel approach using the ’Nintendo Wii’ Balance Board. *Gait Posture*. 2011;33,303–5. doi: 10.1016/j.gaitpost.2010.10.089 21087865

[31] Pessoa TM, Coutinho DS, Pereira VM, Ribeiro NPdO, Nardi AE, Silva ACdOe. The Nintendo Wii as a tool for neurocognitive rehabilitation. training and health promotion. *Computers in Human Behavior*, 2014;31,384–392. doi: 10.1016/j.chb.2013.10.025

[32] Huurnink A, Fransz DP, Kingma I, van Dieën JH. Comparison of a laboratory grade force platform with a Nintendo Wii Balance Board on measurement of postural control in single-leg stance balance tasks. *J Biomech*. 2013;46,1392–5. doi: 10.1016/j.jbiomech.2013.02.018 23528845

[33] Negus JJ, Cawthorne D, Clark R, Negus O, Xu J, March PL, et al. Validity and reliability of the Nintendo Wii Fit Stillness score for assessment of standing balance. *Asia Pac J Sports Med Arthrosc Rehabil Technol*. 2018;15,29–34. doi: 10.1016/j.asmart.2018.09.001 30581757 PMC6300417

[34] Clark RA, Mentiplay BF, Pua YH, Bower KJ. Reliability and validity of the Wii Balance Board for assessment of standing balance: A systematic review. *Gait Posture*. 2018;61,40–54. doi: 10.1016/j.gaitpost.2017.12.022 29304510

[35] Horiuchi K, Imanaka K, Ishihara M. Measuring postural sway during quiet standing: Utilization of Nintendo Wii Balance Board. *IEICE Technical Report*, 2017; HIP 2017–59.

[36] Shingai M, Niijima Ren, Kobayashi Y, Murayama A, Miyadera A, Mukai S. Quantitative evaluation of subjective posture recognition by physiotherapists using a 3D motion capture. *J Phys Ther Sci*. 2020;32(8):510–515. doi: 10.1589/jpts.32.510 Epub 2020 Aug 8. 32884172 PMC7443545

[37] Koltermann JJ, Beck H, Beck M. Investigation of the Correlation between Factors Influencing the Spectrum of Center of Pressure Measurements Using Dynamic Controlled Models of the Upright Stand and Subject Measurements. *Appl*. *Sci*. 2020;10,3741. doi: 10.3390/app10113741

[38] Seno T, Ito H, Sunaga S, Nakamura S. Temporonasal motion projected on the nasal retina underlies expansion-contraction asymmetry in vection. *Vision Res*. 2010;50,1131–9. doi: 10.1016/j.visres.2010.03.020 20371253

[39] Cunningham DW, Nusseck HG, Teufel H, Bulthoff HH. A psychophysical examination of movings, cylindrical virtual reality setups, and characteristic trajectories. IEEE virtual reality conference. 2006;111–118.

[40] Bubka A, Bonato F, Palmisano S. Expanding and contracting optic-flow patterns and vection. *Perception*. 2008;37,704–11. doi: 10.1068/p5781 18605144

[41] Stoffregen TA, Brady BG, Merhi OA, Oullier O. Postural responses to two Techologies for generating optical flow. *Presence Teleoperators & Virtual Environment*. 2014;13,601–615. doi: 10.1109/TCYB.2018.2826016 29994015

[42] Stins JF, Roerdink M, Beek PJ. To freeze or not to freeze? Affective and cognitive perturbations have markedly different effects on postural control. *Hum Mov Sci*. 2011;30,190–202. doi: 10.1016/j.humov.2010.05.013 20727608

[43] Polskaia N, Richer N, Dionne E, Lajoie Y. Continuous cognitive task promotes greater postural stability than an internal or external focus of attention. *Gait Posture*. 2015;41,454–8. doi: 10.1016/j.gaitpost.2014.11.009 25554460

[44] Palmisano S, Arcioni B, Stapley PJ. Predicting vection and visually induced motion sickness based on spontaneous postural activity. *Exp Brain Res*. 2018;236(1),315–329. doi: 10.1007/s00221-017-5130-1 29181555

[45] Takahashi E, OhkiK KimDS. Dissociation and convergence of the dorsal and ventral visual working memory streams in the human prefrontal cortex. *Neuro Image*. 2013:65, 488–498. doi: 10.1016/j.neuroimage.2012.10.002 23063444 PMC4384683

[46] Budisavljevic S, Dell’Acqua F, Castiello U. Cross-talk connections underlying dorsal and ventral stream integration during hand actions. *Cortex*. 2018: 103, 224–239. doi: 10.1016/j.cortex.2018.02.016 29660652

[47] Chowdhury S.A, Takahashi K, Deangelis G. C, Angelaki D. E. Does the middle temporal area carry vestibular signals related to self-motion? *J Neurosci*. 2009;29:12020–12030. doi: 10.1523/JNEUROSCI.0004-09.2009 19776288 PMC2945709

[48] Uesaki M, Ashida H. Optic-flow selective cortical sensory regions associated with self-reported states of vection, *Front Psychol*, 2015;6:775. doi: 10.3389/fpsyg.2015.00775 eCollection 2015. 26106350 PMC4459088

[49] Tohmi M, Meguro R, Tsukano H, Hishida R, Shibuki K. The extrageniculate visual pathway generates distinct response properties in the higher visual areas of mice. *Curr Biol*. 2014;24(6):587–97. doi: 10.1016/j.cub.2014.01.061 24583013

[50] Tabei K, Sato M, Kida H, Kizaki M, Sakuma H, Sakuma H, et al. Involvement of the Extrageniculate System in the Perception of Optical Illusions: A Functional Magnetic Resonance Imaging Study. *PLOS ONE*. 2015;10(6):e0128750. doi: 10.1371/journal.pone.0128750 eCollection 2015. 26083375 PMC4470923

[51] Jang SH, Kwon JW, Yeo SS. Three Dimensional Identification of Medial and Lateral Vestibulospinal Tract in the Human Brain: A Diffusion Tensor Imaging Study. *Front Hum Neurosci*. 2018;12:229. doi: 10.3389/fnhum.2018.00229 eCollection 2018. 29922138 PMC5996120

[52] Barmack NH. Central vestibular system: vestibular nuclei and posterior cerebellum. *Brain Res Bull*. 2003;60(5–6):511–41. doi: 10.1016/s0361-9230(03)00055-8 12787870

[53] Brandt T, Dichgans JM, Koenig E. Differential effects of central versus peripheral vision on egocentric and exocentric motion perception. *Exp Brain Res*. 1973;16,476–491. doi: 10.1007/BF00234474 4695777

[54] Ito H and Shibata I. Self-motion perception from expanding and contracting optical flows overlapped with binocular disparity. *Vision Research*. 2005;45,397–402. doi: 10.1016/j.visres.2004.11.009 15610745

[55] Simpson MJ. Mini-review: Far peripheral vision. *Vision Res*. 2017; 140:96–105. doi: 10.1016/j.visres.2017.08.001 28882754

